# The in-vivo dynamics of *Plasmodium falciparum* HRP2: implications for the use of rapid diagnostic tests in malaria elimination

**DOI:** 10.1186/s12936-022-04245-z

**Published:** 2022-08-03

**Authors:** Louise Marquart, Lachlan Webb, Peter O’Rourke, Michelle L. Gatton, Michelle S. Hsiang, Michael Kalnoky, Ihn Kyung Jang, Henry Ntuku, Davis R. Mumbengegwi, Gonzalo J. Domingo, James S. McCarthy, Sumudu Britton

**Affiliations:** 1 QIMR Berghofer Medical Research Institute, Brisbane, QLD Australia; 2 University of Queensland, Brisbane, QLD Australia; 3 Queensland University of Technology, Brisbane, QLD Australia; 4 Department of Pediatrics, University of Texas, Southwestern, Dallas, TX USA; 5 Malaria Elimination Initiative, Institute for Global Health Services, University of California, San Francisco, CA USA; 6 Department of Pediatrics, University of California, San Francisco, CA USA; 7 Diagnostics Program, PATH, Seattle, WA USA; 8 Multidisciplinary Research Centre, University of Namibia, Windhoek, Namibia

**Keywords:** Rapid diagnostic tests, *Plasmodium falciparum*, Histidine rich protein, Antigen dynamics, Elimination and surveillance

## Abstract

**Background:**

Rapid diagnostic tests (RDTs) that rely on the detection of *Plasmodium falciparum* histidine-rich protein 2 (*Pf*HRP2) have become key tools for diagnosing *P. falciparum* infection. The utility of RDTs can be limited by *Pf*HRP2 persistence, however it can be a potential benefit in low transmission settings where detection of persistent *Pf*HRP2 using newer ultra-sensitive *Pf*HRP2 based RDTs can serve as a surveillance tool to identify recent exposure. Better understanding of the dynamics of *Pf*HRP2 over the course of a malaria infection can inform optimal use of RDTs.

**Methods:**

A previously published mathematical model was refined to mimic the production and decay of *Pf*HRP2 during a malaria infection. Data from 15 individuals from volunteer infection studies were used to update the original model and estimate key model parameters. The refined model was applied to a cohort of patients from Namibia who received treatment for clinical malaria infection for whom longitudinal *Pf*HRP2 concentrations were measured.

**Results:**

The refinement of the *Pf*HRP2 dynamic model indicated that in malaria naïve hosts, *P. falciparum* parasites of the 3D7 strain produce 33.6 × 10^−15^ g (95% CI 25.0–42.1 × 10^−15^ g) of *Pf*HRP2 in vivo per parasite replication cycle, with an elimination half-life of 1.67 days (95% CI 1.11–3.40 days). The refined model included these updated parameters and incorporated individualized body fluid volume calculations, which improved predictive accuracy when compared to the original model. The performance of the model in predicting clearance of *Pf*HRP2 post treatment in clinical samples from six adults with *P. falciparum* infection in Namibia improved when using a longer elimination half-life of 4.5 days, with 14% to 67% of observations for each individual within the predicted range.

**Conclusions:**

The updated mathematical model can predict the growth and clearance of *Pf*HRP2 during the production and decay of a mono-infection with *P. falciparum*, increasing the understanding of *Pf*HRP2 antigen dynamics. This model can guide the optimal use of *Pf*HRP2-based RDTs for reliable diagnosis of *P. falciparum* infection and re-infection in endemic settings, but also for malaria surveillance and elimination programmes in low transmission areas.

**Supplementary Information:**

The online version contains supplementary material available at 10.1186/s12936-022-04245-z.

## Background

Rapid diagnostic tests (RDTs) have become key tools for diagnosing clinical malaria, especially in resource limited settings. These lateral flow antigen capture based assays rely on detection of at least one of three parasite antigen biomarkers: *Plasmodium falciparum* histidine rich protein 2 (*Pf*HRP2), lactate dehydrogenase (LDH), and aldolase. The majority of RDTs used for diagnosis of *P. falciparum* rely on detection of *Pf*HRP2, either alone or in combination with pan-*Plasmodium* LDH (pLDH) [1].

Two major shifts in the approach to malaria control have highlighted gaps in the existing diagnostic toolkit pertaining to RDTs. First, in 2007 the World Health Organization (WHO) in conjunction with the Roll Back Malaria partnerships, and the Bill & Melinda Gates Foundation committed to the ambitious goal of malaria eradication [2]. However, new strategies and tools will be required to achieve this goal. For example, mass or focal screen and treat strategies, rely on the use of sensitive, specific and field ready tests coupled with administration of effective anti-malarial medication [3]. However, data from studies where RDTs have been used for this purpose suggest that they are insufficiently sensitive to achieve the required reduction in cases [4, 5].

Second, in 2010 the WHO Guidelines for the treatment of malaria changed such that parasitological confirmation of infection, using either RDTs or microscopy, was required prior to treatment [6]. However, as with all diagnostic tools, RDTs have their limitations. Importantly *Pf*HRP2 has been shown to persist for up to 36 days after curative treatment [7, 8]. This undermines the feasibility of using *Pf*HRP2-based RDTs to detect re-infection in treated patients in regions of high malaria endemicity [9, 10] and limits their utility in post treatment follow up, for example in pregnant women [11]. Antigen persistence may also delay appropriate treatment for other causes of febrile illness that may be incorrectly attributed to malaria [12–14].

However, persistent antigenaemia is a potential benefit in low transmission settings, where detection of persistent *Pf*HRP2 using newer ultra-sensitive *Pf*HRP2 based-RDTs (usRDT) [15] may provide a measure of recent parasite infection as part of malaria surveillance and elimination programs. While the newer assays do not overcome the problem of false negative results due to *hrp2* gene deletions [16, 17], a better understanding of the dynamics of *Pf*HRP2 during the course of infection, both before and after treatment, is essential to better inform optimal utilization of *Pf*HRP2-based RDTs.

Synthesis of *Pf*HRP2 by *P. falciparum* parasites has been shown, in vitro, to begin within 2 h of initiation of the intraerythrocytic asexual lifecycle, i.e. in early ring stage [18], with the greatest production of *Pf*HRP2 per parasite occurring between ring and trophozoite stages, and absolute production varying in a strain-dependent manner [19]. Although *Pf*HRP2 has been shown to be at highest concentration in the whole blood compared with plasma [20], it has also been suggested that measurement of the plasma level of *Pf*HRP2 may be a useful way to estimate *P. falciparum* biomass compared with measurement of parasitaemia due to sequestration of late stage parasites [21]. Central to the potential use of *Pf*HRP2 to calculate biomass is the clearance half-life of *Pf*HRP2, for which estimates have ranged from 1.10 days [22] up to 4.7 days [23, 24] each of which have been based on limited sampling frequency.

A previous mathematical model of the kinetics of *Pf*HRP2 [25] was developed by simulating the production and clearance of *Pf*HRP2 during a single, relatively synchronous infection of *P. falciparum*. However, the model used quantitative measurement of antigen from only three subjects and utilized key parameter values as reported in the literature. In this study, the mathematical model is refined by using a larger set of samples with more intensive sampling, and the use of a more sensitive assay [26] to better quantify *Pf*HRP2 levels. The utility of the new mathematical model was validated against longitudinal *Pf*HRP2 values measured in a cohort of patients from Namibia who received anti-malarial treatment after mosquito-acquired infection. This mathematical model provides a framework for individualized prediction of *Pf*HRP2 during a mono-infection with *P. falciparum*, improving the understanding of diagnostic marker dynamics to guide the optimal use of *Pf*HRP2-based RDTs.

## Methods

### Study datasets

Two datasets were used in this study. First, data from an induced blood-stage malaria (IBSM) study were used to update the original *Pf*HRP2 mathematical model [25]. Secondly, data from a longitudinal cohort study conducted in Namibia were used to assess performance of the model.

Parasitaemia and *Pf*HRP2 concentration data used to update the original HRP2 mathematical model [25] were from 15 malaria-naïve healthy subjects who participated in an IBSM study (NCT02389348 [27], NCT02431637 [28], NCT02431650 [28], NCT02573857 [29]), conducted by the clinical unit Q-Pharm Pty Ltd at QIMR Berghofer Medical Research Institute between 2015 and 2016. Briefly, subjects were inoculated intravenously on Day 0 with human red blood cells infected with approximately 1800 or 2800 viable 3D7 *P. falciparum*. Parasitaemia was monitored by twice daily blood sampling from Day 4 until treatment on Day 7. Subjects were admitted to the clinical unit, treated with either 200 mg artefenomel/50 mg DSM265, 480 mg piperaquine or 400 mg DSM265 (details in Additional file 1: Material S1), and observed for at least 72 h prior to continuing outpatient monitoring for safety and clearance of all parasites prior to discharge from the study. Model development and analysis were restricted to data corresponding to blood samples collected from Day 4 until Day 11 following inoculation, as this time-period captured the production and decay of the first infection without recrudescence or the appearance of gametocytes.

Data from a longitudinal cohort study were used to validate the updated *Pf*HRP2 model. This study was conducted in the Zambezi Region, Namibia, a low transmission *P. falciparum* dominant setting. Malaria-infected individuals testing positive by RDT [CareStart™ Malaria *Pf*/PAN (HRP-2/pLDH) Antigen, AccessBio] were recruited from health facilities and community test and treat activities. After receiving treatment with artemisinin-based combination therapy (ACT) (artemether–lumefantrine) and low dose primaquine, blood sampling was conducted weekly until testing by the standard RDT and usRDT were both negative for 2 consecutive weeks. A sample of study participants was selected to assess the applicability of the *Pf*HRP2 model to predict *Pf*HRP2 over time after treatment administration. The sample included individuals with a *P. falciparum* mono-infection, confirmed using a PCR assay targeting the *cox3* gene [30] on Day 0 samples; subjects aged between 23 and 27 years were selected to correspond with the interquartile range of the ages of the IBSM study participants. The parasitaemia growth and clearance were simulated for each individual to be used as input into the mathematical model; the body weights of study individuals were simulated from an estimated gender-and-age specific weight curve for Namibian individuals from the 2003/2004 Namibia Household Income and Expenditure Survey [31]. Further detail about the data used for the HRP2 modelling of the Namibia individuals is in Additional file 1: Material S2. As *Pf*HRP2 elimination half-life can differ depending on parasite strain [19] and geographic location [24], and the potential effect of anti-HRP2 antibodies [32], additional modelling was applied to the Namibia sample data using an elimination half-life of 4.5 days as estimated from a study in neighbouring Angola [24].

The IBSM study was approved by the QIMR Berghofer Medical Research Institute Human Research Ethics Committee. The study protocol for the Namibia longitudinal cohort study was approved by the Research Unit of the Ministry of Health and Social Services of Namibia, the University of Namibia Research Ethics Committee and the University of California, San Francisco Committee for Human Research. All participants recruited for the study or their parents/guardians provided written consent.

### Measurement of *Pf*HRP2 concentration and parasitaemia

The concentration of *Pf*HRP2 in each sample was measured using Q-Plex-based enzyme linked immuno-sorbent assays (ELISAs) [15, 26]. These assays are a multiplex array immunoassay (Quansys Biosciences) using a solid phase in defined areas coated with capture antibodies specific for common biomarkers, *Pf*HRP2 and C-reactive protein (CRP). Briefly, for *Pf*HRP2 measurement, a 50 μL sample (whole blood sample and sample diluent of 1:4) was incubated for an hour and after washing away any unbound proteins, a detection mixture containing biotinylated antibody was added and incubated for an hour or three hours. After washing away unbound biotinylated antibody, the plate was incubated with streptavidin–horseradish peroxidase for 20 min. Following the addition of a chemiluminescent substrate, the signal on this array was captured by Q-view imager. *Pf*HRP2 concentration was back calculated from chemiluminescent signals fit to a 5-parameter logistic curve estimating luminescence and *Pf*HRP2 concentration. IBSM samples were measured by 2-plex and Namibia samples by 4-plex assays on this platform.

Parasitaemia for the IBSM participants was measured using a 18s rRNA quantitative PCR (qPCR) assay as previously described [33]. The parasitaemia for individuals from the Namibia study was measured using a qPCR targeting the varATS region of *P. falciparum* [34]. DNA was extracted from thawed packed red blood cells using the Quick-DNATM miniprep kit (Zymo Research Coup) resuspended in 100 µL of water.

### Modelling *Pf*HRP2 kinetics

The original mathematical model [25] assumed the production of *Pf*HPR2 occurred early in the asexual cycle, with all the protein remaining within the infected RBC (iRBC) until the time of schizont rupture, when it is instantaneously released into the circulation. Briefly, the model uses the number of parasites replicating (i.e. schizonts rupturing) at a given time during an infection to estimate the corresponding *Pf*HRP2 concentration. The model assumes that elimination of *Pf*HRP2 is a continuous first-order decay process,

$$d_{k} = \exp \left[ {\frac{{\ln \left( {0.5} \right)k}}{{t_{\frac{1}{2}} }}} \right],$$

where $d_{k}$ is the proportion of *Pf*HRP2 remaining $k$ days after it was produced, and $t_{\frac{1}{2}}$ is the half-life of *Pf*HRP2. The absolute amount of *Pf*HRP2 circulating the body at any day during an infection, $H_{t}$, was calculated using:

$$H_{t} = f\mathop \sum \limits_{j = 1}^{t} d_{t - j} p_{j} ,$$

where $t$ is time in days and $t = 1$ is the first schizogony, $p_{j}$ is the number of replicating parasites circulating in the host on the $j{\text{th}}$ day, and $f$ is the amount of *Pf*HRP2 produced per parasite per lifecycle. Two scenarios for *Pf*HRP2 distribution within the body were considered. The first assumed that *Pf*HRP2 is water soluble and equally distributed throughout the extracellular fluid (ECF). The second assumed that *Pf*HRP2 is distributed in the total blood volume (BV). These two provided the minimum and maximum concentration of circulating *Pf*HRP2, $T_{{min_{t} }}$ and $T_{{max_{t} }}$.

Four aspects of the original model were considered to be updated: the amount of *Pf*HRP2 produced per parasite per life cycle ($f$); the life cycle duration of the *P. falciparum* parasites; individual-specific fluid volumes for the distribution of *Pf*HRP2; and the elimination half-life of *Pf*HRP2 ($t_{\frac{1}{2}}$). These model aspects were updated separately and also in combination. Sensitivity analyses were performed on the final model to assess robustness of the final model to changes in values of $f$ and $t_{\frac{1}{2}}$. Analyses and model fitting were performed in R (Version 3.6.1).

#### Amount of *Pf*HRP2 produced per parasite per life cycle

Following the methodology detailed in Marquart et al. [25], pre-treatment data from the IBSM study were used to calculate an estimate of the amount of *Pf*HRP2 produced per parasite life cycle ($f^{*}$). The original estimate of the amount of *Pf*HRP2 produced, $f_{0}$, was set to 5.2 × 10^−15^ g [19]. This value was adjusted using a scaling factor ($m$). The optimal value of the scaling factor, which produced the minimum residual sum of squares (RSS) between the observed *Pf*HRP2 concentration and the predicted *Pf*HRP2 for $f_{0} \times m$, was calculated for each IBSM individual. The mean scaling factor, $\overline{m}$, was used to determine the new estimate of the amount of *Pf*HRP2 produced per parasite, $f^{*} = f_{0} \overline{m}$, to be utilised in subsequent models.

#### Life cycle duration of the 3D7 strain of *P. falciparum* parasite

The life cycle duration of the 3D7 strain used in the IBSM studies is estimated to be 39 h [35]. To account for the shorter life cycle than the previously assumed 48 h, the model was modified to consider which available time-points would contribute to the peaks of an approximately 40 h life cycle.

#### Body fluid volume

Individualized blood volumes (L) were estimated by using body weight (kg) as, $BV = 0.07$ × weight [36]. Two individualised ECF volumes (L) based on body weight (kg) were considered, the first estimated as $ECF = 0.2$ × weight [37] and the second was gender specific with individualised ECF volumes estimated as $ECF = 0.22$ × weight for females, and $ECF = 0.245$ × weight for males [36].

#### Elimination half-life of *Pf*HRP2 in 3D7 *P. falciparum*

The elimination half-life of *Pf*HRP2 in *P. falciparum* infection was estimated using data measured 12 h apart from treatment administration on Day 7 to Day 11. Estimation of the elimination half-life was restricted to the subset of 10 individuals who had a maximum *Pf*HRP2 concentration exceeding 300 pg/mL. For each individual, the elimination rate $\lambda$, was estimated by fitting a linear regression to the last $n^{*}$ time-points of the natural log *Pf*HRP2 concentration over time with the final data point included in the regression being the first occurrence of the concentration value below the limit of detection (LOD). Five values of $n^{*}$ were considered ranging from 3 to 7. The slope coefficient of the regression model that corresponded to the largest adjusted R-squared for each individual was determined as the elimination rate. The mean elimination rate $\left( {\overline{\lambda } = \frac{1}{10}\mathop \sum \nolimits_{i = 1}^{10} \lambda_{i} } \right)$ was used to estimate the elimination half-life of *Pf*HRP2, by: $t_{\frac{1}{2}} = \frac{\ln \left( 2 \right)}{{\overline{\lambda }}}.$ Regression models were estimated for each subject using the *pkexamine* command within Stata 13.0 (Stata Corporation, College Station, TX).

### Measures to assess model fit

Three metrics were used to evaluate the fit of the model. The first metric is the number of observed *Pf*HRP2 measurements for each subject that were between the minimum and maximum levels of the predicted *Pf*HRP2 ($T_{{min_{t} }}$ and $T_{{max_{t} }}$). The summation over all subjects is calculated as a total number of observed *Pf*HRP2 measurements within the predicted range for the model under consideration. The second metric calculates the residual sum of squares of the model for each subject ($RSS_{i}$), taking the residual of observed log_10_ *Pf*HRP2 concentration from the mean value of the minimum and maximum predicted log_10_ *Pf*HRP2 concentrations. The total RSS (TRSS) is the summation of $RSS_{i}$ over all subjects. The third metric, calculated at the individual level, is the root mean square error (RMSE), calculated as the standard deviation of the residuals from the mean value of the minimum and maximum predicted log_10_ *Pf*HRP2.

## Results

### Model development

**Fig. 1:**
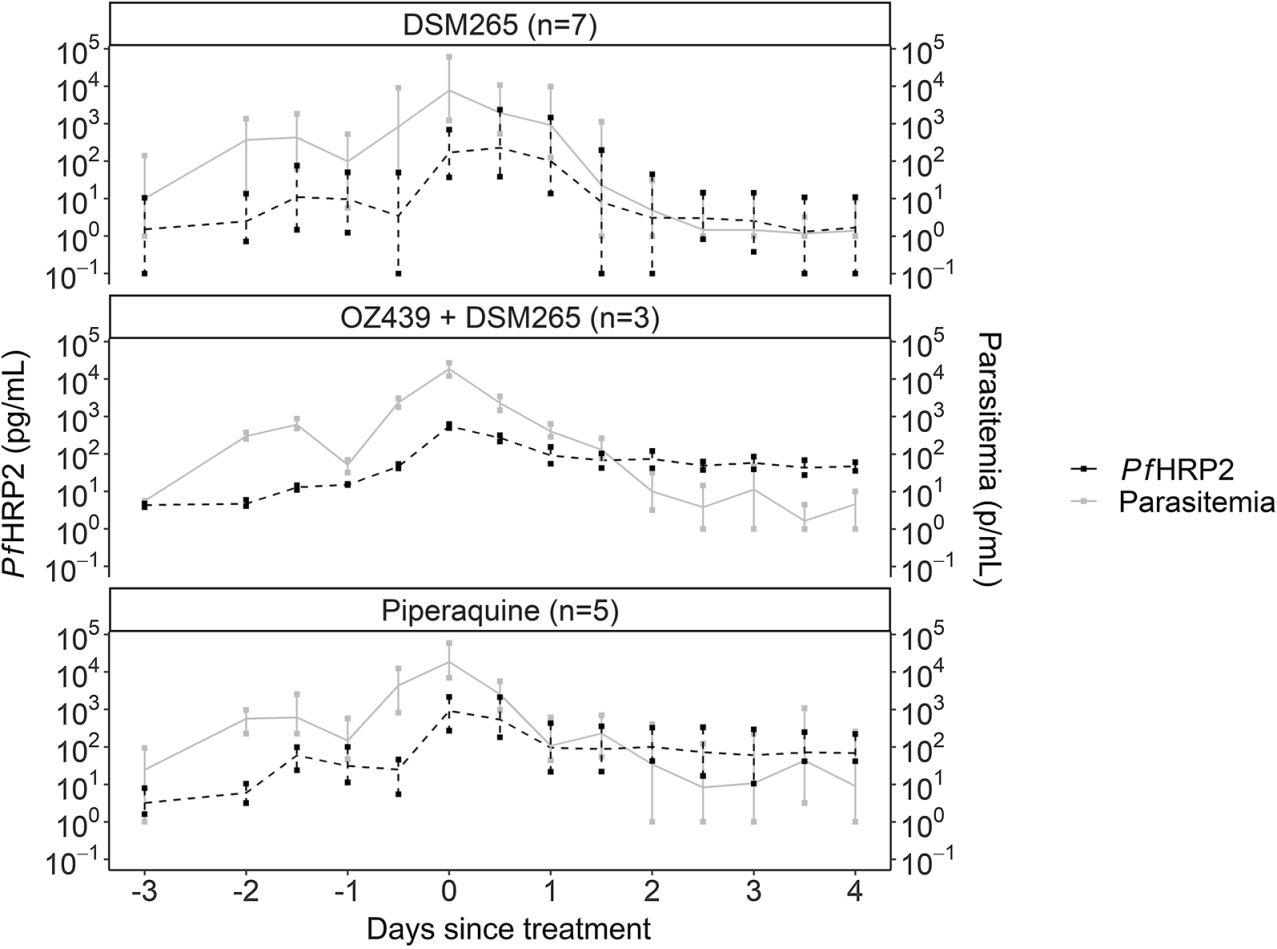
Parasitaemia and *Pf*HRP2 concentration over study period for three anti-malarial treatments in IBSM studies. Relationship between mean parasitaemia (Grey solid line) and mean *Pf*HRP2 concentration (Black dashed line) over the study period used for model development for each of the three anti-malarial treatments in the IBSM study. Bars at each time-point represent minimum and maximum

Demographic data for the 15 individuals from the IBSM study used for model fitting are outlined in Table 1. All individuals were treated with an anti-malarial on Day 7. The majority of individuals were male (11/15, 73%) and the median age was 24 (Interquartile range [IQR]: 23–27) years. The mean weight (range) for females was 68.9 (58.2–75.9) kg and for males was 79.2 (65.0–101.6) kg. During the pre-treatment phase, the median peak parasitaemia (range) was 15,849 (1230–61,357) parasites/mL, and the median peak *Pf*HRP2 concentration (range) was 535 (37–2163) pg/mL. The relationship between parasitaemia and *Pf*HRP2 over the course of the study period for each of the anti-malarial drug groups is summarized in Fig. 1.

**Table 1:** Summary characteristics of the 15 IBSM individuals used for model development

Characteristic	n (%)
Gender	
Male	11 (73%)
Female	4 (27%)
Age	
18–24	9 (60%)
25–29	4 (27%)
30–55	2 (13%)
Weight (kg)	
50–69	4 (27%)
70–89	9 (60%)
90–110	2 (13%)
Pre-treatment peak parasitaemia (parasites/mL)	
0–9999	5 (33%)
10,000–19,999	5 (33%)
20,000–29,999	3 (20%)
30,000–61,357	2 (13%)
Pre-treatment peak *Pf*HRP2 (pg/mL)	
0–299	5 (33%)
300–599	3 (20%)
600–899	4 (27%)
900–2163	3 (20%)
Anti-malarial drug administered at day 7 post inoculation	
200 mg artefenomel + 50 mg DSM265	3 (20%)
480 mg piperaquine	5 (33%)
400 mg DSM265	7 (47%)

### Updating the HRP2 model

The mean scaling factor from the 15 individuals was 6.5 (95% CI 4.8–8.1), corresponding to an estimated amount of *Pf*HRP2 produced per parasite life cycle, f*, of 33.6 × 10^–15^ (95% CI 25.0–42.1 × 10^–15^) g. This value was used in the Base Model, and all subsequent parameters expanded upon this model. The performance of the models considered during the model development process is presented in Table 2. The final model was selected based on model performance as evaluated by the minimum TRSS and the maximum number of points within range.

**Table 2:** Summary of each model aspect evaluated during the development and sensitivity phases, with performance measures

Phase	Model number	Description	TRSS	Number (%) of observations in predicted interval
Development	Base	Previously published model, with updated $f^{*}$ value of 33.6	176.2	27 (13%)
	1	40 h parasite life cycle	185.5	27 (13%)
	2	Body fluid estimated by weight. BV = 0.07 × weight, ECF = 0.2 × weight	171.5	30 (14%)
	3	Body fluid estimated by weight and sex. BV = 0.07 × weight, ECF = 0.245 × weight for males and ECF = 0.22 × weight for females	164.2	37 (18%)
	4	Elimination half-life estimate of 1.67 days	131.1	43 (21%)
	5	Elimination half-life lower bound of 95% CI of 1.11 days	102.7	51 (25%)
	6	Elimination half-life upper bound of 95% CI of 3.40 days	172.7	28 (13%)
	7 (Final)	**Body fluid estimated by weight and sex. BV = 0.07 × weight, ECF = 0.245 × weight for males and ECF = 0.22 × weight for females and elimination half-life of 1.67**	**122.6**	**57 (27%)**
Sensitivity	8	Final model with the upper confidence bound of the 95% CI for $f^{*}$, $f = 42.18$	141.1	49 (24%)
	9	Final model with the lower confidence bound of the 95% CI for $f^{*}$, $f = 24.97$	104.8	64 (31%)
	10	Final model with the upper confidence bound of the 95% CI for half-life, 3.40 days	160.9	37 (18%)
	11	Final model with the lower bound of the 95% CI for half-life, 1.11 days	97.2	57 (27%)

Performance measures evaluated during the development and sensitivity phases were the total residual sum of squares (TRSS) and the number (%) of *Pf*HRP2 observations within the range of the predicted minimum and maximum *Pf*HRP2 concentrations as generated from the models. Model 7 is considered the final model (bold)

The Base Model was similar to the original model of [25] and assumed a 48-h life cycle duration for the 3D7 *P. falciparum* strain, a blood volume of 5 L, an ECF volume of 14 L and a *Pf*HRP2 elimination half-life of 3.67 days. Improvements to the goodness of fit for updates to each of these parameters are given in Table 2. Changing the duration of the life cycle of the 3D7 parasite to 40 h (Model 1, Table 2) did not result in any improvement to the Base Model. Five of the 15 individuals had blood volume estimates based on body weight which were below the previously assumed 5 L (range 4.1–7.1 L) and 14 had sex-weight specific ECF estimates above the previously assumed 14 L (range 12.8–24.9 L). Therefore, incorporating the individualised body fluid calculations based on weight and sex improved the model accuracy (Models 2 and 3, Table 2).

**Fig. 2:**
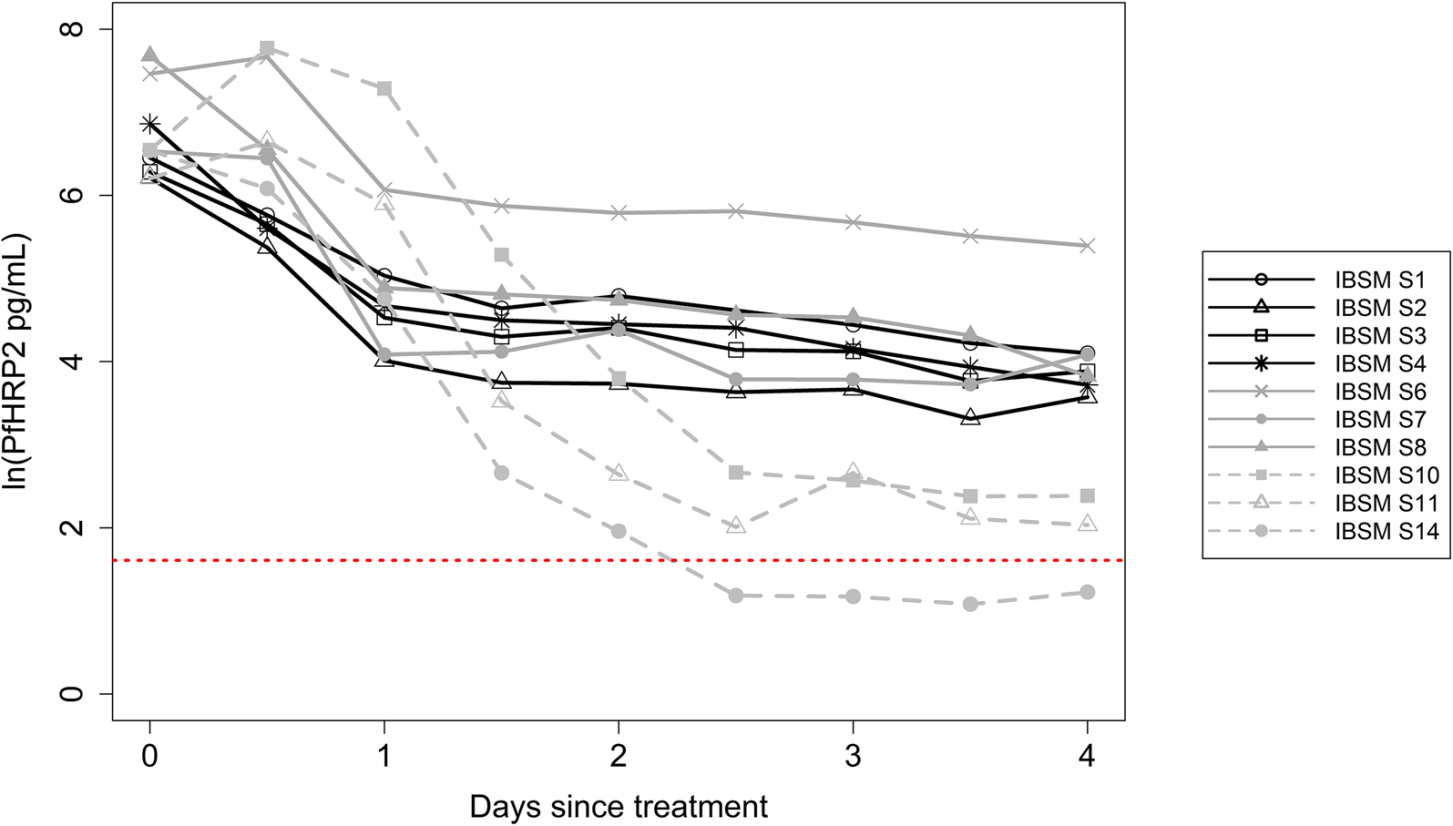
*Pf*HRP2 concentration over time post administration of anti-malarial treatment for estimation of *Pf*HRP2 elimination half-life. *Pf*HRP2 concentration over time post administration of anti-malarial drug for the 10 IBSM individuals with maximum *Pf*HRP2 concentrations exceeding 300 pg/mL that were used to estimate the *Pf*HRP2 elimination half-life. The red dashed line is the reported limit of detection (LOD) for Q-plex ELISA 2-Plex (LOD 5 pg/mL)

The *Pf*HRP2 concentrations over time from the 10 individuals who had maximum *Pf*HRP2 concentrations exceeding 300 pg/mL (Fig. 2) were used to estimate an elimination half-life of 1.67 days (95% CI 1.11–3.40 days) and used to update the models (Models 4–6, Table 2). The performance of the HRP2 model improved with the updated elimination half-life estimates of 1.67 days and the lower confidence interval limit of 1.11 days, with respectively 21% or 25% of observed *Pf*HRP2 concentrations within the predicted range of *Pf*HRP2 concentrations (Table 2).

**Fig. 3:**
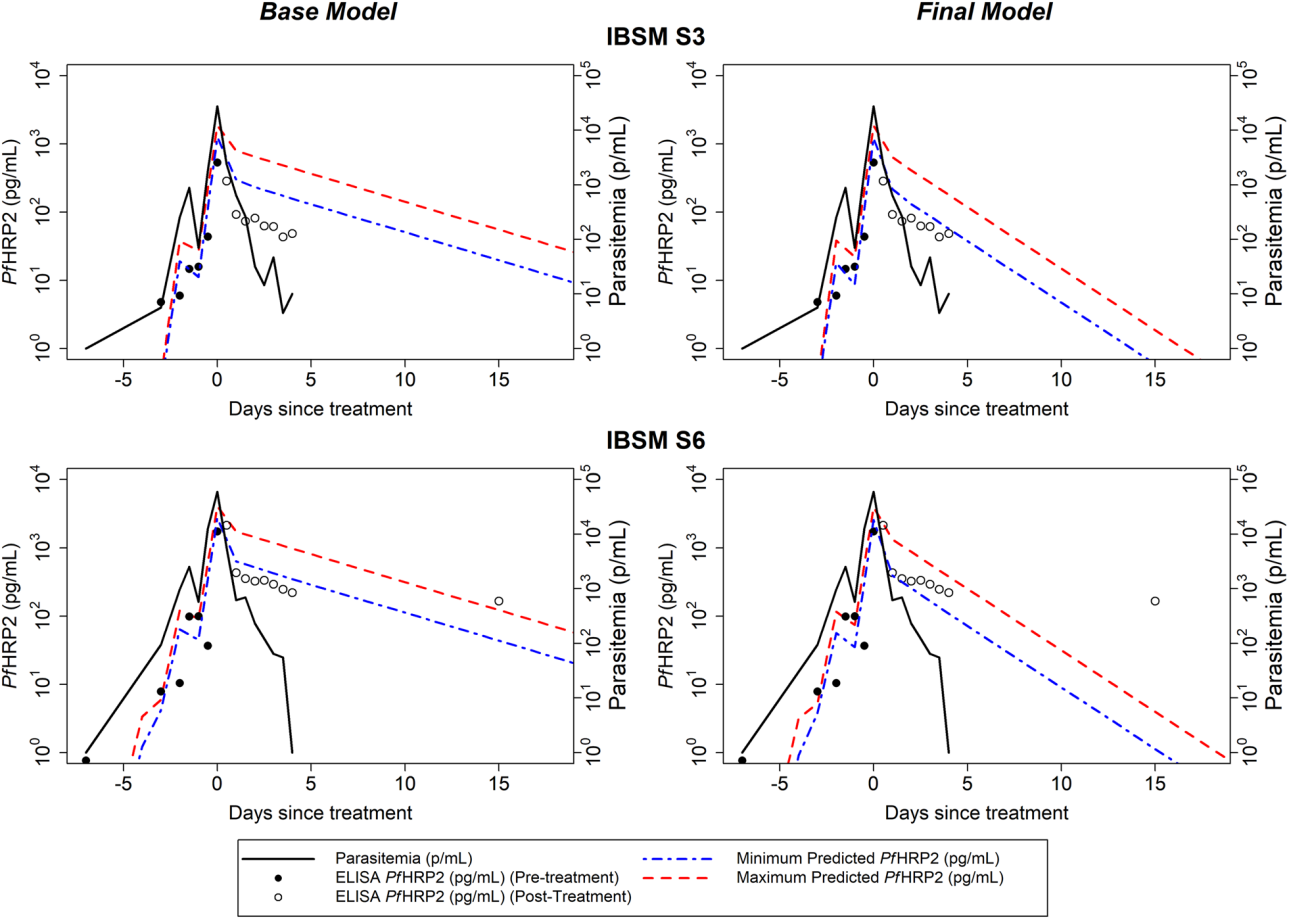
Example fits of the Base Model and the Final Model for two IBSM individuals. The observed parasitaemia over the course of infection is represented by black solid line, observed *Pf*HRP2 concentration is represented by circles (pre-treatment in solid circles and post-treatment in open circles), and the predicted minimum and maximum *Pf*HRP2 concentration from the model is shown as blue and red dashed lines, respectively

A combined model was considered to assess the simultaneous impact of incorporating sex-weight specific estimates of body fluid volumes and updating the elimination half-life of 1.67 days (Model 7, Table 2). The combined model substantially improved the accuracy of the model in comparison to the Base Model, increasing the proportion of observed *Pf*HRP2 concentrations within the predicted range of *Pf*HRP2 concentrations from 13 to 27%. This model utilising the sex-specific weight in the calculation of the ECF volume performed best and was considered the Final Model. The model fits from the Base Model and Final Model for two individuals are shown in Fig. 3 and model fits for all 15 individuals are shown in Additional file 1: Table S1 and Fig. S1.

Sensitivity of the Final Model to changes in key parameters was assessed by considering different models whereby the assumed amount of *Pf*HRP2 produced per parasite ($f^{*}$) and the *Pf*HRP2 elimination half-life estimates ($t_{\frac{1}{2}}$) varied based on the estimated lower and upper bounds of the 95% confidence intervals (Models 8–11, Table 2). The model was sensitive to the changes in $f^{*}$, with the use of the lower bound of the confidence interval for $f^{*}$ of 24.97 × 10^–15^ g improving the model accuracy to 31% of *Pf*HRP2 observations within the predicted range (Model 9, Table 2). The Final Model was also sensitive to the elimination half-life, with a smaller magnitude of TRSS but having the same model accuracy when the upper bound of $t_{\frac{1}{2}}$ of 1.11 days was used (Model 11, Table 2).

### Application of *Pf*HRP2 model to clinical samples from Namibia

The Final Model was applied to the simulated parasitaemia growth and clearance data from a total of six individuals from the Namibia study that met the selection criteria for the validation study. The six individuals all had symptomatic *P. falciparum* mono-infection and were recruited from health facilities before receiving treatment. The details of the 6 individuals are shown in Table 3. The sample comprised of an equal number of males and females aged 24 or 25 years. The estimated weights of the individuals extracted from the sex-and-age specific curves [31] ranged from 56.5 to 62 kg. Parasitaemia at time of enrolment ranged from 99,500 to 49,346,000 parasites/mL, and *Pf*HRP2 concentrations ranged from 2313 to 1.1 × 10^10^ pg/mL. The follow up duration ranged from 21 to 84 days.

**Table 3:** Namibia study participant characteristics and performance of *Pf*HRP2 model with half-life of 4.5 days

ID	Characteristics						Model performance						
	Age	Sex	Estimated weight (kg)	Enrolment parasitaemia (p/mL) × 10^6^	Enrolment *Pf*HRP2 (pg/mL) × 10^6^	Follow up duration (days)	Number (%) observations in predicted interval	RSS	RMSE	Predicted days above 800 pg/mL	Last day observed above 800 pg/mL	Predicted days above 80 pg/mL	Last day observed above 80 pg/mL
F1	24	Male	61	9.46	0.83	84	2 (14%)	10.77	0.877	44.9	56	59.9	63
F2	25	Male	62	2.88	0.33	63	5 (50%)	1.56	0.395	34.3	35	49.3	42
F3	25	Female	58	0.10	0.002	21	1 (25%)	0.41	0.320	14.1	14	29.1	–
F4	24	Male	61	22.61	0.40	56	6 (67%)	1.43	0.399	47.7	42	62.7	–
F5	25	Female	58	0.39	3.66	28	3 (60%)	5.57	1.055	23.3	21	38.3	–
F6	24	Female	56.5	49.35	10,931.8	77	4 (31%)	19.3	1.217	54.9	56	69.9	70

Participant characteristics and model performance of the updated model with *Pf*HRP2 half-life estimate of 4.5 days applied to 6 individuals aged between 23 and 27 years from the study in Namibia in participants with *P. falciparum* mono-infection

*RSS* residual sum of squares, *RMSE* root mean standard error calculated on the mid-point of the minimum and maximum predicted *Pf*HRP2 concentrations—no observations were observed below 80 pg/mL so the last day above the threshold of 80 pg/mL was not able to be reported

**Fig. 4:**
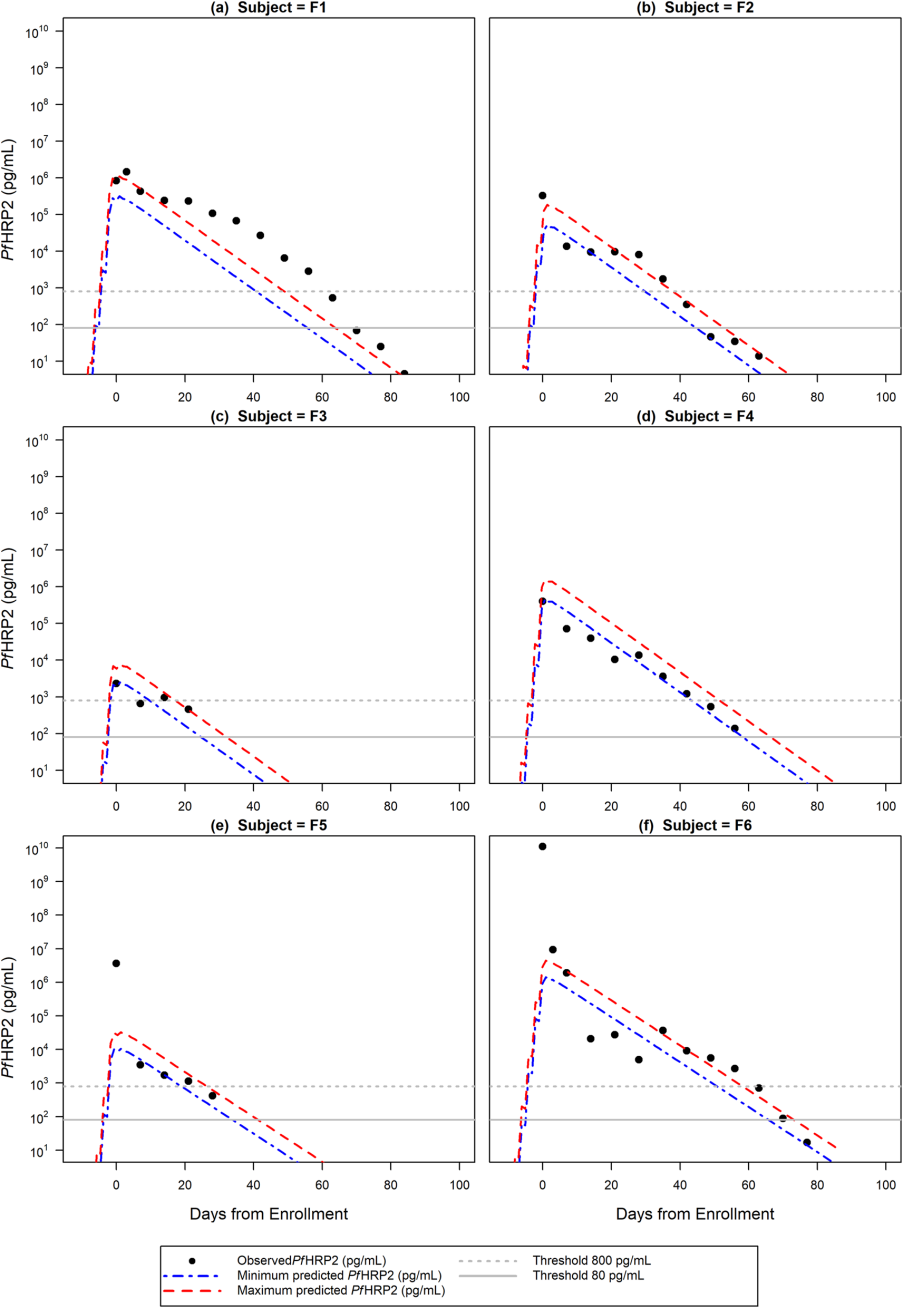
Fits of the final model with elimination half-life of 4.5 days to the Namibia study. Fits of the final *Pf*HRP2 model with elimination half-life of 4.5 days to the 6 individuals from the Namibia study, with observed *Pf*HRP2 concentration represented by closed circles, and minimum and maximum predicted *Pf*HRP2 concentration represented by the blue and red dashed lines, respectively. The dashed grey horizontal line represents the threshold of 800 pg/mL and the solid grey horizontal line represents the threshold of 80 pg/mL which correspond to the positivity threshold of an RDT and usRDT, respectively

The predicted amount of circulating *Pf*HRP2 ($H_{{min_{t} }}$ and $H_{{max_{t} }}$) estimated using the Final Model based on the 3D7 IBSM estimated elimination half-life of 1.67 days underestimated the observed *Pf*HRP2 clearance (Additional file 1: Table S3, Figs. S2, S3) in the Namibian individuals. When the model was modified to use an elimination half-life of 4.5 days, as reported in neighbouring Angola [24], the trend of *Pf*HRP2 clearance over time was better captured with 14% to 67% of observed *Pf*HRP2 concentrations for each individual within the minimum and maximum predicted *Pf*HRP2 concentrations (Table 3, Fig. 4).

Using a limit of detection (LOD) of 800 pg/mL for the standard HRP2-based RDT [1], the model predicted that standard RDT positivity would continue for 14 to 54 days and usRDT would remain above its LOD of 80 pg/mL [15] for 29 to 69 days following study enrolment. The number of days *Pf*HRP2 was predicted to be above 800 pg/mL was underestimated for 3 of the 6 individuals, and the difference in the predicted and observed time above 800 pg/mL ranged between − 6 and 11 days. For the 3 individuals that were observed to have *Pf*HRP2 below 80 pg/mL during the study, the model predicted the number of days above the threshold ranged between − 7 and 3 days of that observed.

## Discussion

In this study, a previously published mathematical model [25] was updated using data from 15 subjects with experimental malaria infection to more accurately describe the dynamics of *Pf*HRP2 during a *P. falciparum* mono-infection. The updated model, when applied to clinical samples from six adults with a *P. falciparum* infection in Namibia using a *Pf*HRP2 half-life of 4.5 days as had been previously reported in the region [24], performed well in predicting the concentration changes in *Pf*HRP2 over time. The predicted *Pf*HRP2 concentrations were subsequently used to estimate the duration that standard RDTs and usRDTs would be expected to remain positive in these individuals, thereby providing a framework for interpreting the results of *Pf*HRP2 based RDTs where *Pf*HRP2 persists following treatment.

A key parameter in modelling the dynamics of *Pf*HRP2 antigenaemia was the amount of *Pf*HRP2 produced per parasite life cycle $\left( f \right)$. Using the combination of a sensitive ELISA to measure whole blood concentration to quantitate *Pf*HRP2 and frequent sampling prior to treatment, in this study it was estimated that 33.6 × 10^–15^ (range: 11.3–71.1 × 10^–15^) g of *Pf*HRP2 is produced in vivo per parasite per life cycle. This is approximately sixfold higher than the previously reported median value of 5.2 × 10^–15^ (range: 1.1–13.0 × 10^–15^) g [19], but lower than the estimate of 141 × 10^−15^ g from the original model [25].

Another key parameter for describing *Pf*HRP2 dynamics was the estimated *Pf*HRP2 elimination half-life, which in this study was found to be 1.67 (95% CI 1.11–3.40) days in malaria naïve healthy volunteers undergoing experimental infection. This is shorter than the 3.67 days previously reported in adult patients with a mixture of uncomplicated or severe malaria [23] who had been treated with ACT or quinine depending on the severity of infection with plasma *Pf*HRP2 measured on days 1, 3, 7, 14 and 21 [23]. The plasma half-life of *Pf*HRP2 reported by Hendriksen et al. was 1.10 (95% CI 0.91–1.29) days from a sample of 30 patients [22]. However, this estimate was based on 3 time points (0, 3 and 7 days post treatment). Plucinski et al. also reported a median half-life of *Pf*HRP2, measured by bead-based assay technology, in the peripheral circulation among symptomatic patients after treatment for uncomplicated *P. falciparum* malaria, of 4.5 (IQR 3.3–6.6) days, 4.7 (IQR 4.0–5.9) days and 3 (IQR 2.1–4.5) days in Angola, Tanzania and Senegal, respectively [24]. These were persons from malaria endemic regions under the age of 10 year (Angola and Tanzania) and between 4 and 20 years (Senegal) who had higher parasitaemia at treatment with ACT (100,000–2 × 10^8^ parasites/mL), and were sampled at days 0, 3, 7 and weekly for 28–42 days [24]. Similar characteristics were observed in the adults from the Namibia field study presented here (parasitaemia of 100,000–5 × 10^7^ parasites/mL and artemisinin-based combination treatment).

There are several potential explanations for the differences in *Pf*HRP2 elimination half-life between this IBSM study and others. These include the effects of immunity, type of anti-malarial treatment used and gametocytaemia. Immunity to malaria is known to sustain higher parasitaemia but also accelerate parasite clearance following treatment [38] with differences evident in children compared with adults [39] but it is not known how this impacts *Pf*HRP2 clearance. Subjects in this IBSM study were malaria naïve, therefore, it may be postulated that this could impact on clearance of infection and, as a result, of *Pf*HRP2. In addition, the anti-malarial agent might influence antigen clearance, with some studies suggesting that treatment with ACT may result in faster *Pf*HRP2 clearance [40], more prolonged clearance of *Pf*HRP2 [7] or no difference in clearance [20, 41] compared to non-artemisinin-based combination treatments as used in the IBSM study. Finally, in this IBSM study subjects were sampled more frequently until day 4 post treatment but prior to the development of gametocytes. However, *Pf*HRP2 is known to be produced by gametocytes in a stage-specific manner [42] and gametocytaemia has been associated with false positivity in RDTs based on *Pf*HRP2 and pan-malarial antigens, particularly among patients treated with chloroquine and sulfadoxine–pyrimethamine compared with artesunate [7]. These reasons may contribute to the improvement in the model performance observed for the ACT-treated subjects in the Namibia cohort when the half-life of 4.5 days was used rather than the half-life estimate derived from the IBSM subjects treated with non-artemisinin-based combination treatments. Further research into the factors that contribute to these differences in elimination half-life are essential to further understand the dynamics of *Pf*HRP2.

This study has several limitations. First, the *Pf*HRP2 elimination half-life estimates for the 3D7 strain presented here were determined using ten IBSM individuals and assumes that *Pf*HRP2 elimination was initiated instantaneously after treatment administration. Further, it was assumed that there were no treatment effects, no differences in the parasite clearance rate between the four different non-artemisinin based antimalarial treatments and that the anti-malarial treatment cleared all stages of the parasite. Similar to other studies [24], it was assumed in this study that *Pf*HRP2 is cleared by first order kinetics. However, Reichert et al. found that *Pf*HRP2 clearance following ACT could be best modelled with biphasic clearance kinetics [43]. In addition, splenic pitting following ACT treatment has been suggested as a mechanism that may explain prolonged *Pf*HRP2 persistence despite rapid parasite clearance with artemisinin-based combination treatment [20] and could be postulated to be a contributing factor to the difference in clearance kinetics and the longer clearance half-life in ACT treated patients compared to the shorter *Pf*HRP2 half-life found in this study where the treatments were with non-ACT anti-malarial agents. Further, this study assumed an absence of gametocytaemia for the purpose of developing model parameters. Therefore, if indeed *Pf*HRP2 accumulation is life cycle stage and strain specific or if *Pf*HRP2 half-life varies by geographic location, applicability of this model may be improved with delineation of these specific parameters.

This model has been developed using data from malaria-naïve subjects challenged with a single infection of the 3D7 strain of *P. falciparum* and has been applied to six individuals from Namibia to illustrate the potential use of a model to predict *Pf*HRP2 dynamics. However, further validation is required for predicting *Pf*HRP2 in different low and high transmission epidemiologic settings and in symptomatic and asymptomatic malaria patients. It is anticipated that in clinical cases of malaria, *Pf*HRP2 concentration will be higher and subject to influence by the interplay between host age, splenic function and immunity that influence parasite replication as well. Even under the relatively controlled experimental conditions of IBSM studies, the *Pf*HRP2 elimination rates vary substantially, reflective of the broad range of *Pf*HRP2 to parasite density observed previously in comparison to lactate dehydrogenase [26]. Therefore, future work is needed to explore the kinetics of *Pf*HRP2 during clinical infection, recrudescence, chronic infections, in infections with different strains of *P. falciparum* and following treatment with anti-malarial agents with different parasite clearance efficacy in order to fully elucidate *Pf*HRP2 dynamics.

Nevertheless, our model substantially increases the current understanding of *Pf*HRP2 antigen dynamics. This is crucial for informing optimal use of *Pf*HRP2-RDTs for reliable diagnosis of *P. falciparum* infection and re-infection in high endemic settings. Application of this model to a subset of patients treated with rapidly schizontocidal drugs facilitated estimation of how long *Pf*HRP2 might persist, at an individual level, and be above the detectable threshold for *Pf*HRP2-based RDTs. Such application could assist in determining when *Pf*HRP2-based RDT positivity reflects previous treatment as opposed to re-infection. Reichert et al. also demonstrated a difference in the ratio of *Pf*HRP2 to LDH prior to treatment compared with after treatment suggesting that such differences in dynamics of these two antigens could inform the better application of usRDTs for the differentiation of prolonged clearance of treated infection from re-infection [43]. Expanding the IBSM model to include LDH dynamics could at a first instance inform these observations and better inform the use of usRDTs for differentiating infection from re-infection in high transmission settings. Additionally, developing complementary LDH dynamic models will be critical in context of the emergence of *hrp2/hrp3* deletion, and a growing reliance on RDTs targeting pLDH [44, 45].

The persistence of *Pf*HRP2 and therefore the role of *Pf*HRP2-based RDTs, including usRDTs, in low transmission and pre-elimination settings continues to be defined. This HRP2 model was developed using data from patients with low level parasitaemia and, therefore, provides a framework for further work simulating the levels of parasite density that maintain a detectable level of *Pf*HRP2, depending on the LOD of the RDT and *Pf*HRP2 elimination half-life. How these relate to probability of transmission will help better inform the best use of usRDTs for the purpose of malaria elimination.

Another application of the model may be estimating the parasite biomass associated with severe malaria. Dondorp et al. showed that adults with severe malaria had a tenfold higher sequestered biomass, as calculated based on *Pf*HRP2 level, compared to adults with uncomplicated malaria [23]. In this study, patient specific weight and sex-based calculation of ECF improved the accuracy of the model compared with circulating blood volume alone, which might reflect this sequestration. However, further characterization of the dynamics of biomarker production by sequestered parasites is required to fully elucidate this link.

## Conclusions

This study presenting an updated model of the dynamics of *Pf*HRP2 found that 33.6 × 10^–15^ g of *Pf*HRP2 was produced in vivo per parasite per life cycle and demonstrated a *Pf*HRP2 clearance half-life of 1.67 days in malaria naïve subjects infected with 3D7 *P. falciparum* parasites and treated with non-ACT anti-malarial agents. The performance of the model in predicting clearance of *Pf*HRP2 and likely duration of RDT positivity post artemisinin-based combination treatment of symptomatic malaria demonstrates the importance of understanding the dynamics of diagnostic biomarkers in order to inform optimal use of RDTs.

## Supplementary Information

**Additional file 1: Material S1.** Anti-malarial treatment details for IBSM individuals. **Table S1.** Antimalarial treatment given for the 15 IBSM individuals from four different studies, as identified with the clinical trial name and clinical trial ID. The antimalarial and dose given at day of treatment on Day 7 is detailed for each subject. **Material S2.** Methods to fit *Pf*HRP2 model to the 6 individuals from the Namibia longitudinal cohort study. **Table S2.** Model performance of the Base model and red the Final model for the 15 IBSM individuals. Performance measures were the number (%) of *Pf*HRP2 observations within the range of the predicted minimum and maximum *Pf*HRP2 concentrations, the residual sum of squares (RSS) and the residual mean sum of square (RMSE). **Table S3.** Model performance of the updated model with *Pf*HRP2 half-life estimate of 1.67 days applied to 6 individuals aged between 23 and 27 years from the study in Namibia in participants with *Plasmodium falciparum *mono-infection. Performance measures were the number (%) of *Pf*HRP2 observations within the range of the predicted minimum and maximum *Pf*HRP2 concentrations, the residual sum of squares (RSS), residual mean sum of square (RMSE), the predicted and observed days above 800 or 80 pg/mL. **Figure S1.** Fits of the Base Model and the Final Model for the 15 IBSM individuals. The observed parasitemia over the course of infection is represented by black solid line, observed *Pf*HRP2 concentration is represented by circles (pre-treatment in solid circles and post-treatment in open circles), and the predicted minimum and maximum *Pf*HRP2 concentration from the model is shown as blue and red dashed lines, respectively. **Figure S2.** Simulated parasitemia growth and clearance over the course of the infection for each of the 6 individuals from the Namibia longitudinal cohort study is represented by the black line. The observed parasitemia (parasites/mL) during the study are represented by the blue triangles and the replicating parasites used as input into the *Pf*HRP2 model are represented by open circles. **Figure S3.** Fits of the final model with elimination half-life of 1.67 days to the 6 individuals from the Namibia longitudinal cohort study, with observed *Pf*HRP2 concentration represented by closed circles, and minimum and maximum predicted *Pf*HRP2 concentration represented by the blue and red dashed lines, respectively. The dashed grey horizontal line represents the threshold of 800 pg/mL and the solid grey horizontal line represents the threshold of 80 pg/mL which correspond to the positivity threshold of an RDT and usRDT respectively.

**Additional file 2.** Includes the R codes and required datasets to perform the final HRP2 models as presented in the manuscript. Futher details about the contents of R code and datasets are included in “File Details.txt”.

## Data Availability

The datasets used and analysed during the current study are included in this published article and its Additional files. The R code of the final *Pf*HRP2 model for the IBSM study and the Namibia Cohort Study are included as Additional file 2.

## Abbreviations

ACT: Artemisinin-based combination therapy
BV: Blood volume
CI: Confidence interval
CRP: C-reactive protein
$d_{k}$: Proportion of *Pf*HRP2 remaining $k$ days after it was produced
ECF: Extracellular fluid
ELISA: Enzyme linked immuno-sorbent assay
$f$: Amount of *Pf*HRP2 produced per parasite per lifecycle
$H_{t}$: Predicted absolute amount of *Pf*HRP2 circulating in the body at any day during an infection
IBSM: Induced blood stage malaria
IQR: Interquartile range
iRBC: Infected red blood cell
LDH: Lactate dehydrogenase
LOD: Limit of detection
$$m$$: Scaling factor
*Pf*HRP2: *Plasmodium falciparum* histidine rich protein 2
$p_{j}$: Number of replicating parasites circulating in the host on the $j{\text{th}}$ day
pLDH: Pan-*Plasmodium* lactate dehydrogenase
qPCR: Quantitative polymerase chain reaction
RDTs: Rapid diagnostic tests
RMSE: Root mean square error
rRNA: Ribosomal ribonucleic acid
RSS: Residual sum of squares
usRDT: Ultra-sensitive rapid diagnostic tests
$t_{\frac{1}{2}}$: Elimination half-life of *Pf*HRP2
$T_{{{\text{max}}}}$: Predicted maximum concentration of circulating *Pf*HRP2
$T_{{{\text{min}}}}$: Predicted minimum concentration of circulating *Pf*HRP2
TRSS: Total residual sum of squares
