# Supplementary material for: The in-vivo dynamics of Plasmodium falciparum HRP2: implications for the use of rapid diagnostic tests in malaria elimination
Source: Malar J. 2022 Aug 3;21:233. doi: 10.1186/s12936-022-04245-z (PMC9351188; doi:10.1186/s12936-022-04245-z)
Supplement: Supplementary file 1 — Additional file 1: Material S1. Anti-malarial treatment details for IBSM individuals. Table S1. Antimalarial treatment given for the 15 IBSM individuals from four different studies, as identified with the clinical trial name and clinical trial ID. The antimalarial and dose given at day of treatment on Day 7 is detailed for each subject. Material S2. Methods to fit PfHRP2 model to the 6 individuals from the Namibia longitudinal cohort study. Table S2. Model performance of the Base model and red the Final model for the 15 IBSM individuals. Performance measures were the number (%) of PfHRP2 observations within the range of the predicted minimum and maximum PfHRP2 concentrations, the residual sum of squares (RSS) and the residual mean sum of square (RMSE). Table S3. Model performance of the updated model with PfHRP2 half-life estimate of 1.67 days applied to 6 individuals aged between 23 and 27 years from the study in Namibia in participants with Plasmodium falciparum mono-infection. Performance measures were the number (%) of PfHRP2 observations within the range of the predicted minimum and maximum PfHRP2 concentrations, the residual sum of squares (RSS), residual mean sum of square (RMSE), the predicted and observed days above 800 or 80 pg/mL. Figure S1. Fits of the Base Model and the Final Model for the 15 IBSM individuals. The observed parasitemia over the course of infection is represented by black solid line, observed PfHRP2 concentration is represented by circles (pre-treatment in solid circles and post-treatment in open circles), and the predicted minimum and maximum PfHRP2 concentration from the model is shown as blue and red dashed lines, respectively. Figure S2. Simulated parasitemia growth and clearance over the course of the infection for each of the 6 individuals from the Namibia longitudinal cohort study is represented by the black line. The observed parasitemia (parasites/mL) during the study are represented by the blue triangles and the replicating para [file 12936_2022_4245_MOESM1_ESM.pdf]

Additional File 1

Manuscript Title: The in-vivo dynamics of *Plasmodium falciparum* HRP2: implications for the use of rapid diagnostic tests in malaria elimination

Louise Marquart[1], Lachlan Webb[1], Peter O'Rourke[1], Michelle Gatton[2], Michelle S. Hsiang[3,4,5], Michael Kalnoky[6], Ihn Kyung Jang[6], Henry Ntuku[4], Davis R. Mumbengegwi[7], Gonzalo J Domingo[6], James S. McCarthy*[1,8], Sumudu Britton*[1, 8]

[1]QIMR Berghofer Medical Research Institute, Brisbane, QLD, Australia

[2]Queensland University of Technology, Brisbane, QLD, Australia

[3]Department of Pediatrics, University of Texas, Southwestern, Dallas, TX, USA

[4]Malaria Elimination Initiative, Institute for Global Health Services, University of California, San Francisco, CA, USA

[5]Department of Pediatrics, University of California, San Francisco, CA, USA

[6]Diagnostics Program, PATH, Seattle, WA, USA

[7]Multidisciplinary Research Centre, University of Namibia, Windhoek, Namibia

[8]University of Queensland, Brisbane, QLD, Australia

*****Joint last authors

This additional file contains:

Material S1: Anti-malarial treatment details for IBSM individuals

Table S1: Antimalarial treatment given for the 15 IBSM individuals from four different studies, as identified with the clinical trial name and clinical trial ID. The antimalarial and dose given at day of treatment on Day 7 is detailed for each subject.

Material S2: Methods to fit *Pf*HRP2 model to the 6 individuals from the Namibia longitudinal cohort study

Table S2: Model performance of the Base model and red the Final model for the 15 IBSM individuals. Performance measures were the number (%) of *Pf*HRP2 observations within the range of the predicted minimum and maximum *Pf*HRP2 concentrations, the residual sum of squares (RSS) and the residual mean sum of square (RMSE).

Table S3: Model performance of the updated model with *Pf*HRP2 half-life estimate of 1.67 days applied to 6 individuals aged between 23 and 27 years from the study in Namibia in participants with *Plasmodium falciparum* mono-infection. Performance measures were the number (%) of *Pf*HRP2 observations within the range of the predicted minimum and maximum *Pf*HRP2 concentrations, the residual sum of squares (RSS), residual mean sum of square (RMSE), the predicted and observed days above 800 or 80 pg/mL.

Figure S1: Fits of the Base Model and the Final Model for the 15 IBSM individuals. The observed parasitemia over the course of infection is represented by black solid line, observed *Pf*HRP2 concentration is represented by circles (pre-treatment in solid circles and post-treatment in open circles), and the predicted minimum and maximum *Pf*HRP2 concentration from the model is shown as blue and red dashed lines, respectively.

Figure S2: Simulated parasitemia growth and clearance over the course of the infection for each of the 6 individuals from the Namibia longitudinal cohort study is represented by the black line. The observed parasitemia (parasites/mL) during the study are represented by the blue triangles and the replicating parasites used as input into the *Pf*HRP2 model are represented by open circles.

Figure S3: Fits of the final model with elimination half-life of 1.67 days to the 6 individuals from the Namibia longitudinal cohort study, with observed *Pf*HRP2 concentration represented by closed circles, and minimum and maximum predicted *Pf*HRP2 concentration represented by the blue and red dashed lines, respectively. The dashed grey horizontal line represents the threshold of 800 pg/mL and the solid grey horizontal line represents the threshold of 80 pg/mL which correspond to the positivity threshold of an RDT and usRDT respectively.

Material S1: Treatment details for IBSM individuals

The 15 malaria-naïve health subjects used to update the original HRP2 mathematical model were selected from four IBSM studies (NCT02389348 (1), NCT02431637 (2), NCT02431650 (2), NCT02573857 (3)) conducted by QIMR Berghofer Medical Research Institute between 2015 and 2016 at the clinical unit Q-Pharm Pty Ltd. Briefly, subjects were inoculated intravenously on Day 0 with human red blood cells infected with approximately 1800 or 2800 viable 3D7 *P. falciparum*. Parasitemia was monitored by twice daily blood sampling from Day 4 until treatment on Day 7. Subjects were admitted to the clinical unit, treated with an antimalarial agent as detailed in Table S1. Subjects IBSM S1- S3 were from the OZ/DSM study (NCT02389348) and were treated with 200mg artefenomel and 50mg DSM265. Subjects IBSM S4 and S5 from the EFITA study (NCT02431637) and subjects IBSM S6-S8 from the OZGAM study (NCT02431650) were treated with 480mg of piperaquine on Day 7. Subjects IBSM S9 – S15 from the DSMOZ-2 study were treated with 400mg of DSM265 (NCT02573857). Data used in the *Pf*HRP2 modelling includes parasitemia and *Pf*HRP2 data collected twice-daily from Day 4 to Day 11.

Table S1: Antimalarial treatment given for the 15 IBSM subjects from four different studies, as identified with the clinical trial name and clinical trial ID. The antimalarial and dose given at day of treatment on Day 7 is detailed for each subject.

ID	Clinical Trial Name	Clinical Trial ID	Antimalarial (Day 7)	Antimalarial Dose	Inoculum dose
IBSM S1<br>IBSM S2<br>IBSM S3	OZ439/DSM265 (1)	NCT02389348	artefenomel /DSM265	200mg artefenomel + 50mg DSM265	1800
IBSM S4<br>IBSM S5	EFITA (2)	NCT02431637	piperaquine	480mg	2800
IBSM S6<br>IBSM S7<br>IBSM S8	OZGAM (2)	NCT02431650	piperaquine	480mg	2800
IBSM S9<br>IBSM S10<br>IBSM S11<br>IBSM S12<br>IBSM S13<br>IBSM S14<br>IBSM S15	DSMOZ-2 (3)	NCT02573857	DSM265	400mg	2800

Material S2: Methods to fit *Pf*HRP2 model to the 6 individuals from the Namibia longitudinal cohort study

Methodology to simulate parasitemia dynamics

The parasitemia growth and clearance were simulated for each of the six individuals from the study in Namibia. The parasite growth was simulated from a sine-wave growth model (4) with parameter estimates derived from Equation S1 applied to 177 IBSM subjects inoculated with 3D7 as detailed in Wockner et al. 2020 (5). Parasitemia growth for individual i and time j, denoted Y_{ij} (parasites/mL), was simulated as:

$$\log_{10}(\mathrm{Y_{ij}}) = (a + b_{0i}) + (m + b_{1i}) \times t + \; c \times \sin\left(\frac{2\pi t}{p} + (k + b_{2i})\right) \qquad \text{Equation S1}$$

where t is days from initial infection and it is assumed that a = y-intercept set to 0, m = parasite growth rate per day set to 0.758, c = sine-wave amplitude set to 0.645, k = sine-wave phase shift set to 6.34 following Wockner et al. 2020 (5), and p = duration of the parasite life-cycle set to 2 days. Individual level random effects for a, m and k denoted by b_{0i}, b_{1i} and b_{2i}, respectively, were assumed to follow a multivariate normal distribution with zero mean and variance-covariance given by the following matrix: $\begin{pmatrix} 0.116 & 0.001 & 0.051 \\ 0.001 & 0.001 & 0.012 \\ 0.051 & 0.012 & 0.291 \end{pmatrix}$.

The parasitemia growth model was used to simulate parasitemia until the value of the parasitemia recorded at time of study enrolment. Antimalarial treatment was administered at study enrolment and it was assumed that parasite clearance immediately commenced, which followed a first-order exponential decay with a constant clearance rate of 0.3 $\log_{10}$ parasites/mL per day. The simulated parasitemia growth and clearance for each individual with the observed parasitemia densities are shown in Figure S2. The replicating parasites, assumed to occur at the observed peaks during the growth phase and every two days during the clearance phase as represented by open circles in Figure S2, were used as the parasitemia input into the *Pf*HRP2 model.

Methodology to impute body weights

The body weights of the study individuals were not available, and were imputed from an estimated gender-and-age specific weight curve for Namibia individuals (Figure 2, (6)) based on data from 44,230 individuals aged between 0 and 80 years from the 2003-2004 Namibia Household Income and Expenditure Survey (NHIES) and estimated with a non-parametric regression and a local linear approach using the *cnpe* module in the DASP STATA package (7). The imputed weights for each individual are shown in Table 3 and were used to estimate the individualised blood volume and extracellular fluid volume as input in the *Pf*HRP2 model.

Table S2: Model performance of the Base model and the Final model for the 15 IBSM individuals. Performance measures were the number (%) of *Pf*HRP2 observations within the range of the predicted minimum and maximum *Pf*HRP2 concentrations, the residual sum of squares (RSS) and the residual mean sum of square (RMSE).

**ID**	**Base Model**			**Final Model**		
	**Number (%) observations in predicted range**	**RSS**	**RMSE**	**Number (%) observations in predicted range**	**RSS**	**RMSE**
IBSM S1	1 (7.7%)	3.47	0.52	6 (46.2%)	2.47	0.44
IBSM S2	2 (14.3%)	4.49	0.57	3 (21.4%)	2.83	0.45
IBSM S3	2 (14.3%)	6.52	0.68	2 (14.3%)	4.25	0.55
IBSM S4	2 (14.3%)	1.67	0.35	10 (71.4%)	0.85	0.25
IBSM S5	2 (14.3%)	6.80	0.70	3 (21.4%)	5.05	0.60
IBSM S6	2 (14.3%)	3.37	0.49	8 (57.1%)	2.27	0.40
IBSM S7	1 (7.1%)	4.33	0.56	5 (35.7%)	2.41	0.41
IBSM S8	1 (7.1%)	3.73	0.52	3 (21.4%)	2.08	0.39
IBSM S9	3 (21.4%)	16.05	1.07	4 (28.6%)	10.52	0.87
IBSM S10	4 (28.6%)	16.45	1.08	4 (28.6%)	11.41	0.90
IBSM S11	3 (23.1%)	12.64	0.99	1 (7.7%)	8.81	0.82
IBSM S12	2 (14.3%)	17.05	1.10	4 (28.6%)	11.69	0.91
IBSM S13	0 (0%)	28.90	1.44	0 (0%)	19.41	1.18
IBSM S14	1 (7.1%)	20.48	1.21	2 (14.3%)	16.39	1.08
IBSM S15	1 (7.1%)	30.23	1.47	2 (14.3%)	22.19	1.26

Table S3: Model performance of the updated model with *Pf*HRP2 half-life estimate of 1.67 days applied to 6 individuals aged between 23 and 27 years from the study in Namibia in participants with *Plasmodium falciparum* mono-infection. Performance measures were the number (%) of *Pf*HRP2 observations within the range of the predicted minimum and maximum *Pf*HRP2 concentrations, the residual sum of squares (RSS), residual mean sum of square (RMSE), the predicted and observed days above 800 or 80 pg/mL.

ID	Model Performance						
	**Number (%) observations in predicted range**	**RSS**	**RMSE**	**Predicted days above 800 pg/mL**	**Last day observed above 800 pg/mL**	**Predicted days above 80 pg/mL**	**Last day observed above 80 pg/mL**
F1	0 (0%)	545.4	6.24	17.2	56	23.2	63
F2	1 (10%)	180.1	4.24	13.6	35	19.6	42
F3	2 (50%)	7.6	1.38	6.8	14	12.8	-
F4	1 (11%)	104.1	3.40	20.5	42	25.5	-
F5	0 (0%)	22.6	2.12	9.7	21	15.7	-
F6	1 (8%)	314.6	4.92	22.9	56	27.9	70

RSS: Residual Sum of Squares; RMSE: Root Mean Standard Error calculated on the mid-point of the minimum and maximum predicted *Pf*HRP2 concentrations; - No observations below 80pg/mL so the last day above the threshold of 80 pg/mL was not able to be reported.

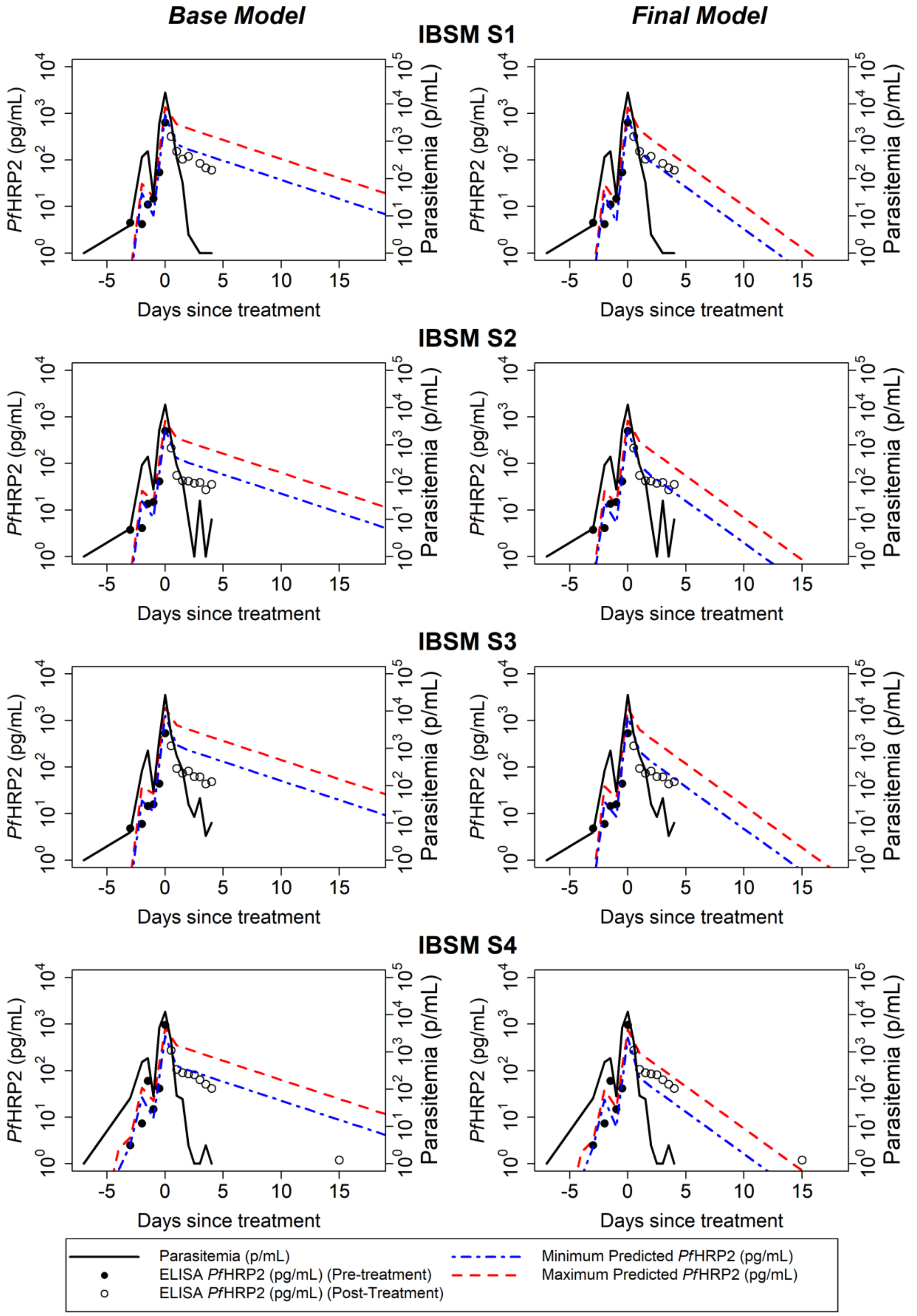
Base Model
Final Model
IBSM S1
IBSM S2
IBSM S3
IBSM S4
PfHRP2 (pg/mL)
Parasitemia (p/mL)
Days since treatment
Parasitemia (p/mL)
ELISA PfHRP2 (pg/mL) (Pre-treatment)
ELISA PfHRP2 (pg/mL) (Post-Treatment)
Minimum Predicted PfHRP2 (pg/mL)
Maximum Predicted PfHRP2 (pg/mL)

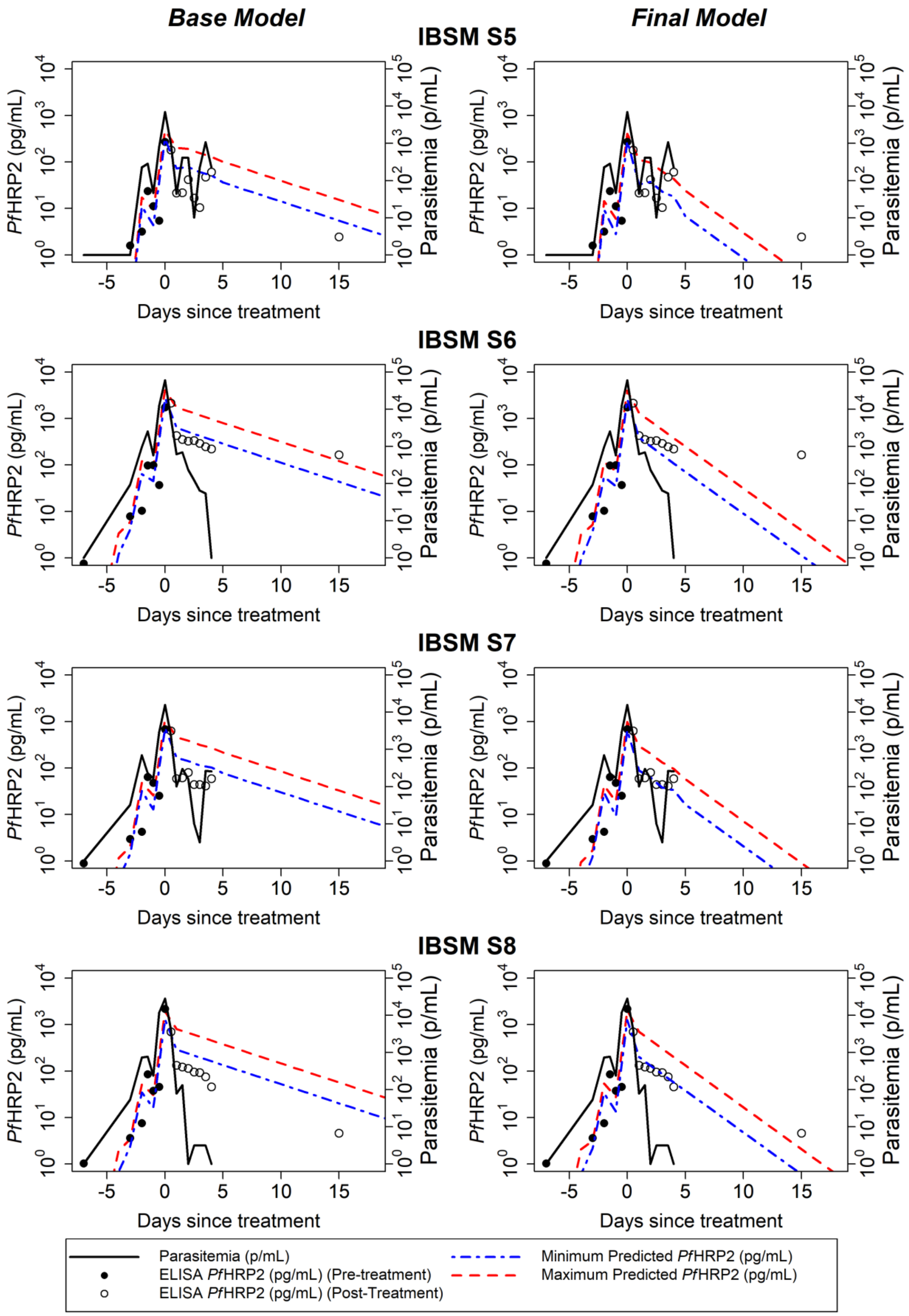
Base Model
Final Model
IBSM S5
IBSM S6
IBSM S7
IBSM S8
PfHRP2 (pg/mL)
Parasitemia (p/mL)
Days since treatment
Parasitemia (p/mL)
ELISA PfHRP2 (pg/mL) (Pre-treatment)
ELISA PfHRP2 (pg/mL) (Post-Treatment)
Minimum Predicted PfHRP2 (pg/mL)
Maximum Predicted PfHRP2 (pg/mL)

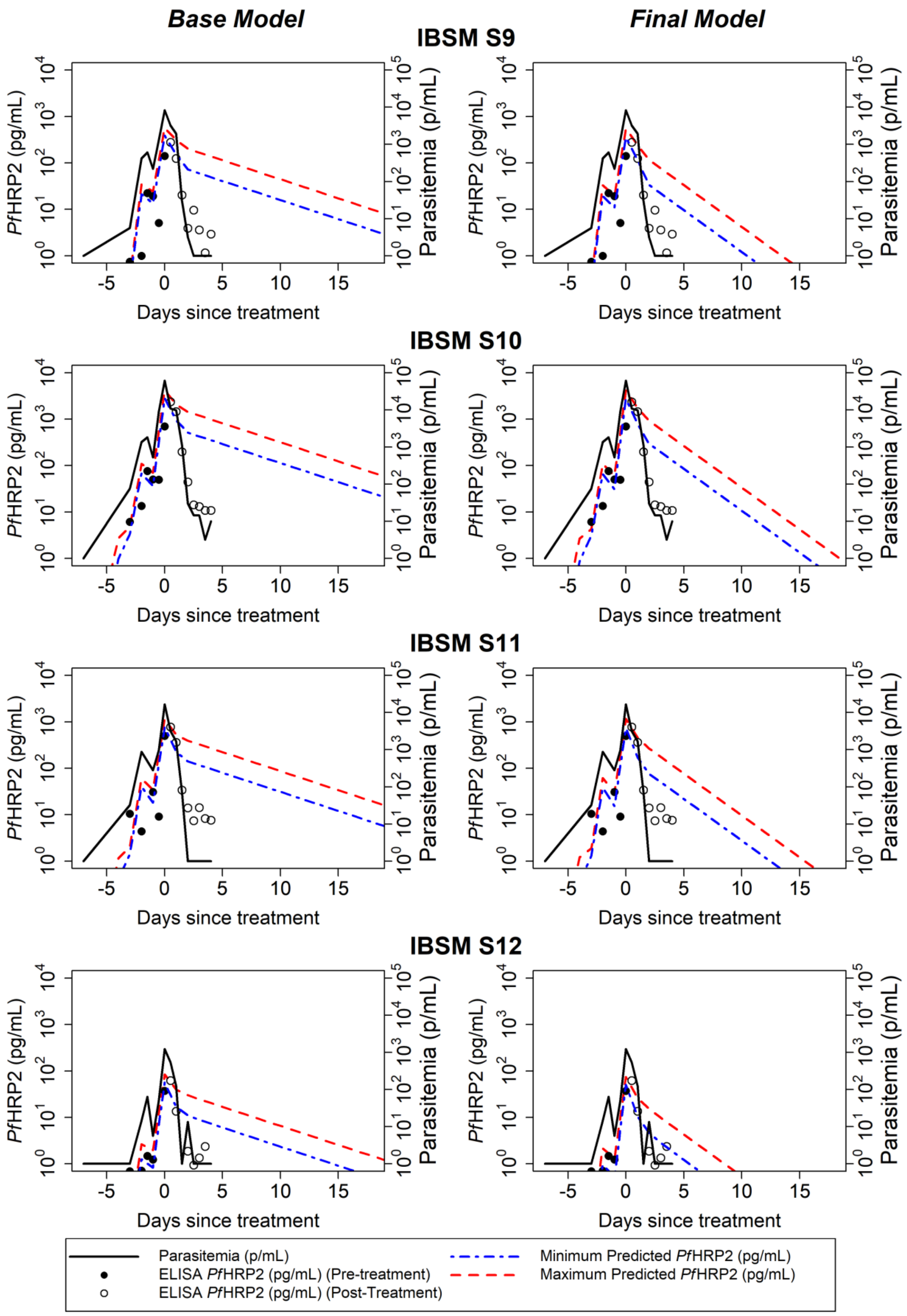

Base Model
Final Model
IBSM S9
IBSM S10
IBSM S11
IBSM S12
PfHRP2 (pg/mL)
Parasitemia (p/mL)
Days since treatment
Parasitemia (p/mL)
ELISA PfHRP2 (pg/mL) (Pre-treatment)
ELISA PfHRP2 (pg/mL) (Post-Treatment)
Minimum Predicted PfHRP2 (pg/mL)
Maximum Predicted PfHRP2 (pg/mL)

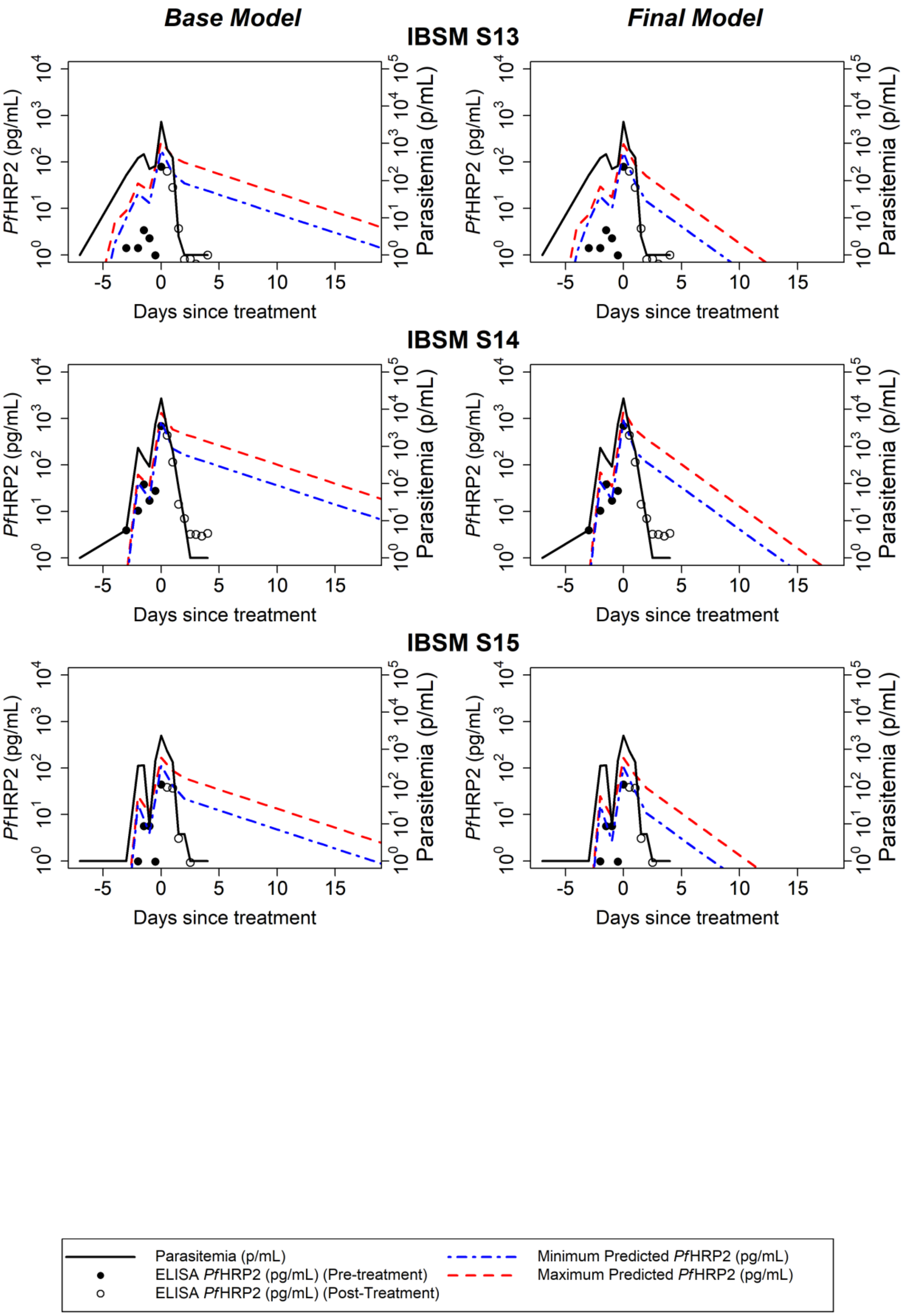

Figure S1: Fits of the Base Model and the Final Model for the 15 IBSM individuals. The observed parasitemia over the course of infection is represented by black solid line, observed *Pf*HRP2 concentration is represented by circles (pre-treatment in solid circles and post-treatment in open circles), and the predicted minimum and maximum *Pf*HRP2 concentration from the model is shown as blue and red dashed lines, respectively.

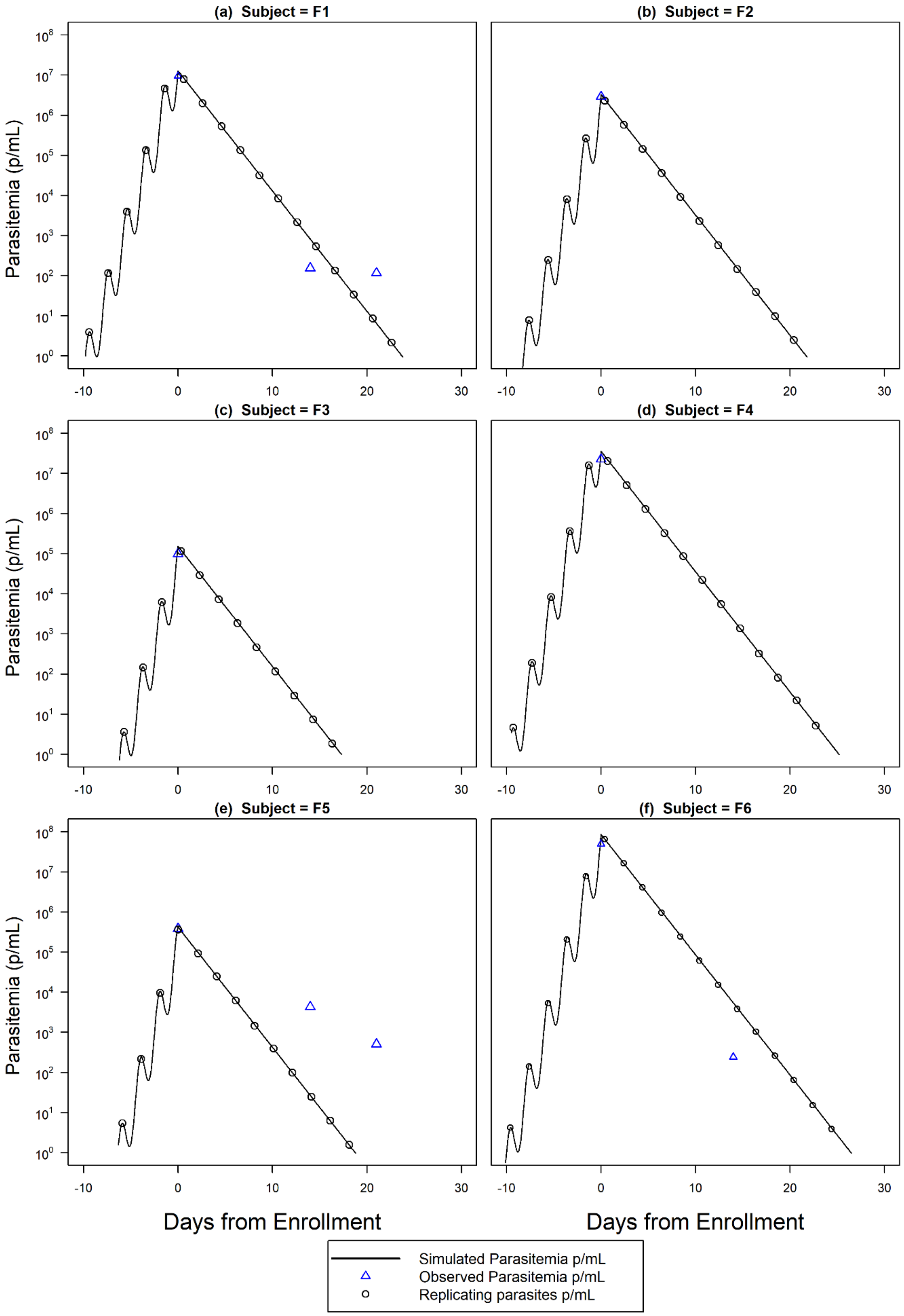

Figure S2: Simulated parasitemia growth and clearance over the course of the infection for each of the 6 individuals from the Namibia longitudinal cohort study is represented by the black line. The observed parasitemia (parasites/mL) during the study are represented by the blue triangles and the replicating parasites used as input into the *Pf*HRP2 model are represented by open circles.

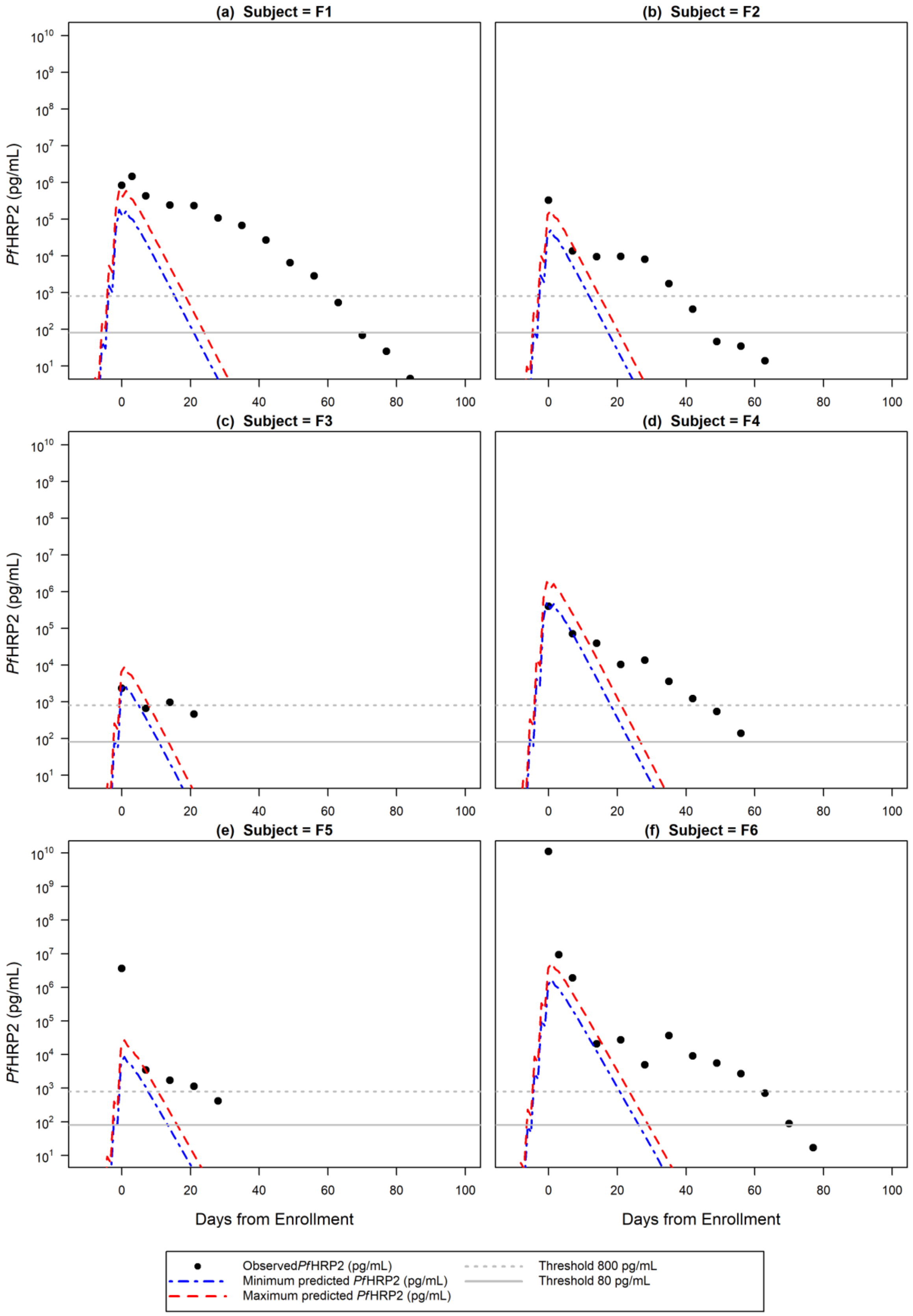

Figure S3: Fits of the final model with elimination half-life of 1.67 days to the 6 individuals from the Namibia longitudinal cohort study, with observed *Pf*HRP2 concentration represented by closed circles, and minimum and maximum predicted *Pf*HRP2 concentration represented by the blue and red dashed lines, respectively. The dashed grey horizontal line represents the threshold of 800 pg/mL and the solid grey horizontal line represents the threshold of 80 pg/mL which correspond to the positivity threshold of an RDT and usRDT respectively.

References

1. McCarthy JS, Ruckle T, Elliott SL, Ballard E, Collins KA, Marquart L, Griffin P, Chalon S, Mohrle JJ. A single-dose combination study with the experimental antimalarials artefenomel and DSM265 To determine safety and antimalarial activity against blood-stage *Plasmodium falciparum* in healthy volunteers. Antimicrob Agents Chemother. 2019;64(1).

2. Collins KA, Wang CY, Adams M, Mitchell H, Rampton M, Elliott S, Reuling IJ, Bousema T, Sauerwein R, Chalon S, Mohrle JJ, McCarthy JS. A controlled human malaria infection model enabling evaluation of transmission-blocking interventions. J Clin Invest. 2018;128(4):1551-62.

3. Collins KA, Ruckle T, Elliott S, Marquart L, Ballard E, Chalon S, Griffin P, Mohrle JJ, McCarthy JS. DSM265 at 400 milligrams clears asexual stage parasites but not mature gametocytes from the blood of healthy subjects experimentally infected with *Plasmodium falciparum*. Antimicrob Agents Chemother. 2019;63(4).

4. Simpson JA, Aarons L, Collins WE, Jeffery GM, White NJ. Population dynamics of untreated *Plasmodium falciparum* malaria within the adult human host during the expansion phase of the infection. Parasitology. 2002;124(3):247-63.

5. Wockner LF, Hoffmann I, Webb L, Mordmuller B, Murphy SC, Kublin JG, O'Rourke P, McCarthy JS, Marquart L. Growth rate of *Plasmodium falciparum*: analysis of parasite growth data from malaria volunteer infection studies. J Infect Dis. 2020;221(6):963-72.

6. Araar A, Levine S, Duclos J-Y. Body mass index, poverty and inequality in Namibia. Paper prepared for the Central Bureau of Statistics, Namibia 2009.

7. Araar A, Duclos J-Y. DASP: Distributive analysis STATA package. PEP, World Bank, UNDP and Université Laval.; 2007.
